# Management and Treatment of the Insane in Texas

**Published:** 1888-04

**Authors:** A. N. Denton

**Affiliations:** Late Superintendent State Lunatic Asylum, Austin, Texas


DANIEL'S

### TEXAS MEDICAL JOURNAL.

PUBLISHED MONTHLY.—SUBSCRIPTION $2.00 A YEAR.

Vol. 3. AUSTIN, APRIL, 1888. No. 10.

By unanimous vote of the members, this Journal was made the Official Organ of the Austin District Medical Society.

### ORIGINAL ARTICLES.

CONTRIBUTED EXCLUSIVELY TO THIS JOURNAL.

*The Articles in this Department are accepted and published with the understanding that we are not responsible for, nor do we indorse the views and opinions of the writers by so doing.*

### MANAGEMENT AND TREATMENT OF THE INSANE IN TEXAS.

*By A. N. Denton, M. D., late Superintendent State Lunatic Asylum, Austin, Texas.*

Read at Austin District Medical Society, March 8, 1888, and ordered published in the OFFICIAL ORGAN.

MR. PRESIDENT and Gentlemen of the Society—The subject of this paper is not, strictly speaking, a medical subject. The author does not intend at this time to discuss the causes, pathology, or medical treatment of the insane, but to discuss briefly the management and treatment of this unforunate class of our defective population in this State, the said treatment here having no medical significance.

While the subject is not one of medicine or surgery, it is nevertheless a legitimate and proper subject, from a humanitarian standpoint, for consideration and discussion by the members of this society, and by the medical profession of Texas.

Inasmuch as I am not now connected with any institution for the care of the insane, and have no intention or desire to be, in the future, I feel at liberty to express my views and opinions, without subjecting myself to the criticism of writing from interested motives. I deem it my duty as a citizen of Texas, and as a member of the medical profession, to write plainly, and to call your attention to the wretched policy of our State government during its past history, and point out what I conceive to be the remedy for some of the evils, which have been encountered in the past in the management of the insane. I have no time or space to detail to you the interesting history of the progress which has been made in the management and treatment of the insane in every civilized government of Europe and America. Progress in this direction has kept pace with improvement in other branches of science.

Improvement in the treatment of the insane began about the middle of the eighteenth century. About this time Bedlams, or houses of detention for the insane began to be established in some of the cities of Europe. The motives, however, for the establishment of these institutions were not altogether philanthropic or on account of sympathy for the inmates, but there is reason to believe that these measures were adopted for the public safety and comfort. Nor did these measures bring about a termination of the barbaric period of the treatment of the insane. The houses, misnamed asylums, were under little or no supervision, and were, in fact, merely prisons of the very worst description.

The unhappy inmates of these so-called asylums were immured in cells, chained to the walls or floors, flogged, starved and frequently killed. It it almost incredible, but nevertheless true, that this unhappy condition existed far into the present century.

Conolly says, there is clear proof of the continued existence of these abuses in 1828, and it cannot be denied that not a few of them remained in some public and private asylums in 1850.

The names of Pinel in France, and Luke in England, are indissolubly connected with the history of reform in the management and treatment of the insane, and to their efforts must be ascribed the awakening, not only of the public, but of the medical profession to the true principles of management. But many years elapsed before these humane teachings were universally accepted. It was not until 1836 that the mechanical restraints were entirely abolished in an English hospital for the insane. This took place at Lincoln, where Dr. Goodwin Hill abolished all engines of restraint. Shortly afterwards Conolly adopted the same policy, and many other institutions followed the humane example, until at this time, I believe, more than half of the asylums for the care of the insane in Europe and America have adopted this policy, and most of them, that continue the use of restraints, have reduced them now to a very few exceptional cases.

The ancient idea that the insane were "possessed of evil spirits" or devils, is long ago exploded, and instead, it is universally admitted, that insanity is but the manifestation through the nervous system, of pathological changes in the brain, or other parts of the physical system, and for its cure demands rational treatment, as in other ailments of the human body.

With this and other improvements in the knowledge of pathology and treatment, have come others, and still greater in management. As before stated, the old idea that force and restraint were generally necessary, has also passed away, and in its stead the heavenly law of kindness is the universal rule, in all well managed institutions.

*Mechanical restraints*, in my judgment, are never justifiable in the management of the insane, and should never be employed, except for the following reasons, viz.: 1st, to prevent self injury or suicide. 2d, to prevent serious bodily injury of others. 3d, for surgical purposes, to prevent the removal of dressings of wounds, etc. The greatest liberty should be allowed, consistent with the safety of the patient, and those with whom he is associated. Means and ways have been devised and adopted for employment and recreation, and briefly, in the best conducted institutions for the care and treatment of this unhappy class, the constant unremitting effort of the management is in all possible ways to make the inmates comfortable, and their surroundings home-like and pleasant. It has been found from abundant experience that such measures are not only called for in the interests of humanity, but are also conducive to cure.

These are some of the leading principles and ideas which should be kept constantly in view by the superintendent in his management of the insane.

In order to succeed, certain pre-requisites are necessary. He should be fitted by temperament and education for the trust.

No man should ever be permitted to assume the care of the insane, who has not the power at all times and under all circumstances, to control his own passions and impulses,.

A man who is tempted to reply in kind to the abusive language of a man or woman whose mind is unbalanced, or who is capable of entertaining prejudice against such patients, is totally unfit for the management or care of the insane in any capacity; A harsh word or action should never be indulged in or allowed.

In addition to these attributes, experience in the management is of the greatest importance. This is so obvious that it would scarcely seem necessary to assert it, but it would seem from the history of the oldest and largest asylum of any state, that the fact was unknown, or has been wilfully disregarded. Experience is important, not only in the details of the management, but also in the medical treatment of the patients. Who does not recognize the importance of a knowledge of the peculiarities and idiosyncrasies of patients in private practice, and the advantages resulting from adhering as long as possible to the family physician in the treatment of disease? The principle is so well known, and universally recognized by all classes of people that I need not adduce detailed arguments in its support. But if experience is necessary in each individual case or family, in private practie, how much more so must it be in the management, and medical treatment of six or seven hundred insane people?

I state but fact, which is well known to those who have had experience in the management of such institutions, that it requires at least one year in an Asylum of this magnitude, for an industrious, energetic superintendant, moving about among his patients every day, to learn the names of the inmates, and to recognize them when he meets them.

To study and learn the physical and mental peculiarities of each, requires, of course, a much longer experience; but I assume that no one, or at all events, no medical man in Texas will dispute the importance of experience in the treatment and the management of the insane. The principle is so well recognized, and its importance is so well known that in nearly all the states of the Union, changes in the management are never made, except for good and sufficient cause.

In many of the states the superintendents have held their positions for more than a quarter of a century, and the great improvement in the management of these institutions, and the treatment of their unfortunate inmates, bears the strongest testimony to the wisdom of the policy.

I assert, and confidently believe, that the abuses and mismanagement, which unfortunately have at times, characterized the history of our own Institution, as well as the lack of progress and improvement in the general management, have been mainly due, and are still due to the frequent changes in the internal management. The Asylum has beenclaimed, and regarded as a chattel, belonging to each incoming governor; and to the shame of the State, has generally been conferred upon a favorite, as a reward for political service, without regard to the qualifications or fitness of the applicant:

The following brief history of the changes effected by successive governors, in the management of the Asylum near this city, teaches an important lesson, which the people of Texas should learn, in the interest of the unfortunates confined there, whose wretchedness and miseries under the most favorable circumstances, are scarcely paralleled in the long catalogue of human ailments.

The act, creating the State Lunatic Asylum was approved August 28, 1856, and took effect November 2, of same year.

Dr. J. C. Perry, the first Superintendent, was appointed May 27, 1857, by Governor E. M. Pease. An appropriation of $50,000 was made by the legislature to carry into effect the act of August 28, 1856, by the erection of suitable buildings.

On the 13th of February, 1858, Dr. Charles Kunow was appointed superintendent, by Governor Hardin R. Runnels, vice Dr. Perry removed. Cause not assigned. About this time a suit at law was instituted by Dr. Perry against Dr. Kunow, for the possession of the office of superintendent, which finally reached the Supreme Court, and was there decided in favor of Kunow. (See 26 Texas Report.) Less than two years later the superintendency was again changed, by the appointment or Dr. B. Graham to the office, on the 9th of January, 1860, by Governor Sam Houston. No reason assigned for the change. A little more than a year later, on the 1st of April, 1861, Dr. C. G. Kunow was again appointed superintendent by Governor Ed. Clark, vice Dr. Graham removed. No cause given for the change. It was due, however, to the secession of the State from the Federal Union, and the consequent deposition of General Houston from the office of Governor.

Dr. Kunow held the position until November 1861, when he was succeeded by Dr. Josephus M. Steiner. The administration of Dr. Steiner extended from the date of his appointment, to November 9, 1865, which at that time, was the longest period that any superintendent had held the office, and the longest since that time, with the exceptions of that of the writer, and Dr. D. R. Wallace. The incumbency of the author extended over a period of a little more than four years, and that of Dr. Wallace over a period of nearly five years.

Dr. B. Graham was appointed to succeed Dr. Steiner on the 9th of September, 1865, by Military Governor A J. Hamilton, and continued to administer the affairs of the asylum until he was succeeded by Dr. W. P. Beall, who was appointed by Governor Throckmorton, on the 20th of August, 1866. Dr. Beall was superintendent one year and three days, and was removed on the 23d of August, 1867, by Governor E. M. Pease, and Dr. B. Graham was, for the third time appointed, in his stead.

On the 27th of March, 1870, Dr. Graham was again removed, and Dr. James Corley was appointed in his stead by Governor E. J. Davis. In less than one year from this date Dr. Corley, shared the fate of his predecessors, and was succeeded by Dr. Weissenberg, who was appointed by Governor Davis on the 1st of March, 1871. Dr. Weissenberg was removed on the 10th of February, 1874, and was succeeded by Dr. D. R. Wallace, who was appointed by Gov. Richard Coke.

On the 19th, of April, 1879, the administration was again changed, by the retirement of Dr. Wallace and the appointment of Dr. W. E. Saunders, by Gov. O. M. Roberts.

On the 14th, of May, 1881, Dr. Saunders retired, and was succeeded by Dr. L. J. Graham, who was also appointed by Gov. Roberts, on the 20th, of Jan. 1883. Dr. Graham was succeeded by the writer of this article, who was appointed by Gov. Jno. Ireland. About this time the 18th Legislature, seeing the evil resulting from, and the pernicous effect of such frequent changes of the internal management of the Institution, passed an Act taking away from the Governor the power to appoint the superentendent, and making it the duty of the Board of Managers to elect to that office. It was hoped and believed that this Act would, in a measure, remove the management from the baneful influence of party politics, and thus secure a permanent government for the Institution. Under this law the writer was elected to a second term by the Board of Managers. At the end of his second term, the Board again indicated their intention to re-elect the writer, but were prevented from doing so by Gov. L. S. Ross, who had selected Dr. J. S. Dorset for the position. The old board refused to elect Dr. Dorset, was removed, and a new board appointed, who carried out the wishes of the Governor, by the election of the present incumbent, who assumed the duties of the position on the 5th, of Feb. 1887.

The recent history of the Institution is so well known that I need not advert to it. All these changes were made without the assignment of a cause, or even the preferment of a single charge, so far as the record shows.

It will be seen from the foregoing brief history that during the thirty years, since the erection of the Asylum, there have been no less than fifteen separate and distinct administrations of its affairs. Its history consists of little else but a succession of changes by the appointment of new saperintendents with each change in the executive office of the State.

I doubt if there is another institution of this character in this, or any other country, that can present such a remarkable series of changes of administration in the institution above recited, involving not only a change of superintendent, but almost the entire working force of the Asylum. As soon as a new governor is installed the Asylum is placed in the hands of strangers without experience, and untried in the delicate duties of treating and caring for the insane.

It seems strange, that an official of such exalted dignity and power, should be so unmindful of the welfare of six hundred human beings, or, on the other hand, so ignorant of the wants, necessities and interests of a great institution of this character, as to wilfully place it in the hands of strangers, who, whatever may be their disposition or capacities, are unfit for the charge, for want of experience in the care of the insane. Doubtless some of these changes were judiciously and properly made; but a majority of the superintendents received their commission as a reward for political services, without regard to merit or fitness for the trust. As above stated, the Eighteenth Legislature attempted to remove the institution from the influence of party politics, and to place it in the hands of a board of managers, whose intimate relations with the asylum, and ample opportunity to observe its internal management, rendered its members especially well qualified to supervise its affairs and to appoint its resident officers. But it has proven practically inadequate to protect the institution, which for more than a quarter of a century, has been periodically bartered, and transferred, with as little scruple as a trader would transfer a herd of bee f cattle.

In the interest of more than six hundred of the most helpless and deeply afflicted of the human race ; in the name, and in the interests of the people of Texas, whose friends are lodged there, I appeal to this Society, and to the medical profession of Texas, to use its great influence with the law-making power of the State, to establish for our asylums a permanent government, which may only be changed "for good and sufficient reason."

Our unfortunates who are confined there, are unable to utter their complaints. They are beyond the pale of law. Their voices cannot be heard. It is our especial duty, though unsolicited, to use all honorable efforts to procure the passage of an act, removing this great charity from the influence and power of the politician and place it in the hands of a board of managers who shall be held responsible for the welfare of the institution, and who may only be removed for good and sufficient cause.

Until this is done the wretched inmates of a great institution, founded and endowed by our fathers for their care and protection, will continue to be in the future, as in the past, bartered and transferred like sheep in the market, and will continue to be at the mercy of inexperienced and unqualified persons, incapable of giving them such treatment as their condition requires and demands.

